# Fluconazole-Induced Ploidy Change in *Cryptococcus neoformans* Results from the Uncoupling of Cell Growth and Nuclear Division

**DOI:** 10.1128/mSphere.00205-17

**Published:** 2017-06-14

**Authors:** Sophie Altamirano, Diana Fang, Charles Simmons, Shreyas Sridhar, Peipei Wu, Kaustuv Sanyal, Lukasz Kozubowski

**Affiliations:** a Department of Genetics and Biochemistry, Clemson University, Clemson, South Carolina, USA; b Molecular Biology and Genetics Unit, Molecular Mycology Laboratory, Jawaharlal Nehru Centre for Advanced Scientific Research, Bangalore, India; Carnegie Mellon University

**Keywords:** antifungal resistance, cytokinesis, heteroresistance, ploidy, yeasts

## Abstract

*Cryptococcus neoformans* is a pathogenic yeast that causes lethal cryptococcal meningitis in immunocompromised patients. One of the challenges in treating cryptococcosis is the development of resistance to azole antifungals. Previous studies linked azole resistance to elevated numbers of copies of critical resistance genes in aneuploid cells. However, how aneuploidy is formed in the presence of azole drugs remains unclear. This study showed that treatment with inhibitory concentrations of an azole drug, fluconazole (FLC), resulted in a significant population of cells with increased DNA content, through the following defects: inhibition of budding, premature mitosis, and inhibition of cytokinesis followed by replication in the mother cell. Inhibition of and/or a delay in cytokinesis led to the formation of cells with two or more daughter cells attached (multimeric cells). To investigate which part of cytokinesis fails in the presence of FLC, the dynamics of the actomyosin ring (AMR), septins, and Cts1, a protein involved in cell separation, were analyzed with time-lapse microscopy. Following the constriction of the AMR, septins assembled and the septum was formed between the mother and daughter cells. However, final degradation of the septum was affected. Enlarged cells with aberrant morphology, including multimeric cells, exhibited an increased potential to proliferate in the presence of FLC. These findings suggest that pleiotropic effects of FLC on growth and mitotic division lead to an increase in DNA content, resulting in cells less sensitive to the drug. Cells with increased DNA content continue to proliferate and therefore increase the chance of forming resistant populations.

**IMPORTANCE** Azoles are antifungals that are widely utilized due to relatively low toxicity and cost of treatment. One of their drawbacks, however, is that azoles are primarily cytostatic, leaving fungal cells capable of developing drug resistance. The human pathogen *Cryptococcus neoformans* acquires resistance to the azole drug fluconazole (FLC) through the development of aneuploidy, leading to elevated expression of key resistance genes, a mechanism that is also common for *Candida albicans* (K. J. Kwon-Chung and Y. C. Chang, PLoS Pathog 8:e1003022, 2012, https://doi.org/10.1371/journal.ppat.1003022; J. Morschhäuser, J Microbiol 54:192–201, 2016, https://doi.org/10.1007/s12275-016-5628-4). However, the exact ways in which FLC contributes to increased resistance in either of these important fungal pathogens remain unclear. Here we found that FLC treatment leads to an increase in DNA content in *C. neoformans* through multiple mechanisms, potentially increasing the size of a pool of cells from which aneuploids with increased resistance are selected. This study demonstrated the importance of FLC’s inhibitory effects on growth and cytokinesis in the generation of cell populations with decreased sensitivity to the drug.

## Abstract (precis)

Azoles are antifungals that are widely utilized due to relatively low toxicity and cost of treatment. One of their drawbacks, however, is that azoles are primarily cytostatic, leaving fungal cells capable of developing drug resistance. The human pathogen *Cryptococcus neoformans* acquires resistance to the azole drug fluconazole (FLC) through the development of aneuploidy, leading to elevated expression of key resistance genes, a mechanism that is also common for *Candida albicans* (K. J. Kwon-Chung and Y. C. Chang, PLoS Pathog 8:e1003022, 2012, https://doi.org/10.1371/journal.ppat.1003022; J. Morschhäuser, J Microbiol 54:192–201, 2016, https://doi.org/10.1007/s12275-016-5628-4). However, the exact ways in which FLC contributes to increased resistance in either of these important fungal pathogens remain unclear. Here we found that FLC treatment leads to an increase in DNA content in *C. neoformans* through multiple mechanisms, potentially increasing the size of a pool of cells from which aneuploids with increased resistance are selected. This study demonstrated the importance of FLC’s inhibitory effects on growth and cytokinesis in the generation of cell populations with decreased sensitivity to the drug.

## INTRODUCTION

Genomic integrity is a crucial attribute of all living forms, and yet, under some circumstances, transient abnormalities in chromosomal number and composition are beneficial for the cell population (1, 2). For instance, development of aneuploidy is a common survival mechanism for cells under the selective pressure of adverse environmental conditions (2). Aneuploidy is typical for malignant cells that strive to proliferate despite nutrient limitation and inhibitory effects of anticancer drugs (3). Aneuploidy occurs frequently in pathogenic organisms that encounter harsh inhibitory conditions within the host and battle against therapeutic regimens; prominent examples include the protozoan *Leishmania*; an ascomycetous yeast, *Candida albicans*; and a basidiomycetous yeast, *Cryptococcus neoformans*, the most common causal agent of fungal meningitis in AIDS and transplant patients (4, 5–8). How can changes in chromosomal composition lead to improved survival? The key to this process is the selection favoring cells with elevated numbers of copies of specific genes that provide a growth advantage under particular inhibitory conditions. In most cases, retention of an entire additional chromosome or addition of an arm of a specific chromosome occurs in selected population of cells. Importantly, the environmental inhibitory effects not only select for organisms with specifically altered chromosomal composition but also often induce genomic changes by mechanisms that remain ill defined (1). The resulting pool of cells with various karyotypes increases the chances of survival; individuals with beneficial chromosomal compositions are selectively favored under conditions of inhibitory pressure (1). Prominent examples of fungal pathogens developing resistance to azole antifungal treatments are those observed with *C. albicans* and *C. neoformans*. The fungistatic azole drug fluconazole (FLC) targets lanosterol 14α-demethylase (Erg11), which is responsible for converting lanosterol to ergosterol in the ergosterol biosynthesis pathway (9). Ergosterol is known to be an essential component for cell membrane permeability and fluidity, but the exact inhibitory consequences of ergosterol diminishment remain elusive (10).

Specific genes that confer resistance to FLC and thus determine the selection of a particular chromosomal configuration in *C. albicans* and *C. neoformans* are well established (4, 7). On the other hand, the mechanisms through which FLC potentially leads to changes in chromosomal composition in these human pathogens of significance remain poorly characterized. In a diploid yeast, *C. albicans*, treatment with FLC leads to growth inhibition, premature nuclear and spindle cycles, and a failure in cytokinesis resulting in formation of multimeric cells (multimeras) containing tetraploid nuclei (11). Tetraploid cells give raise to aneuploid populations; most of the fit individuals of those populations are selected under FLC inhibitory pressure, leading to FLC resistance (11). The exact mechanism through which FLC inhibits cytokinesis in *C. albicans* remains unknown.

It is well established that the form of *in vitro* FLC-triggered drug resistance in *C. neoformans* called heteroresistance is a relatively rare occurrence and that its occurrence is based primarily on the formation of aneuploids (<1% of the FLC-treated population) (8). However, the initial effects of FLC on the population of *C. neoformans* have not been characterized and the mechanisms by which FLC triggers changes in chromosome number in *C. neoformans* remain unclear. Recent studies reveal several differences between *C. neoformans* and *Saccharomyces cerevisiae* with respect to mitosis, which may suggest that the mechanism of FLC-dependent aneuploidy formation in *C. neoformans* may differ from previously described mechanisms in hemiascomycetous yeasts, including *C. albicans* (12).

Here we investigated the mechanisms through which FLC affects ploidy in *C. neoformans*. Our data suggest that exposure to inhibitory concentrations of FLC leads to a progressive diminishment of the ability to initiate budding and subsequent growth while permitting nuclear events. In addition, FLC inhibits cytokinesis, most likely by imposing a delay or a permanent block in the degradation of the primary septum. The resulting populations of cells with an increase in DNA content grow better in the presence of the inhibitory concentration of FLC, hypothetically increasing the chance of forming the aneuploids from which the most highly adapted cells are selected, giving rise to fully resistant populations.

## RESULTS

### FLC treatment results in an increase in DNA content in a significant fraction of cells.

**FIG 1:**
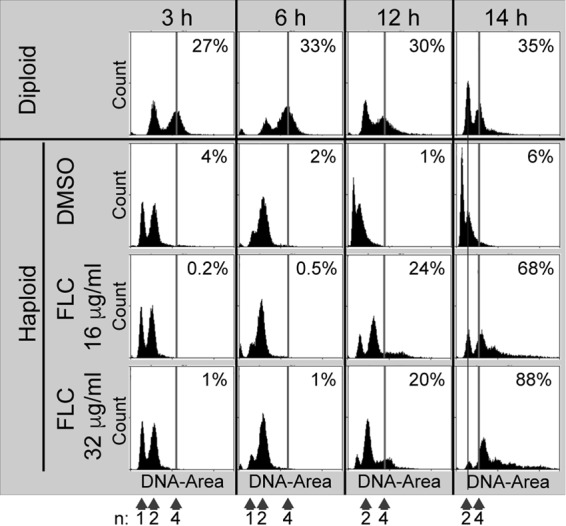
FLC treatment results in an increase in ploidy in a significant fraction of cells. *C. neoformans* cells treated with FLC were fixed, stained with propidium iodide (PI), and examined using fluorescence flow cytometry. Treatment of cells with 32 μg/ml FLC at 24°C for 12 and 14 h resulted in ~20% and 88%, respectively, of the cell population showing ploidy levels at or above 4N, as indicated by the vertical line.

At the concentration of FLC that is equal to the heteroresistance level, resistant colonies are aneuploids, initially with disomic chromosome 1 (8). FLC could potentially act solely as a selection agent to allow growth of only a small fraction of the preexisting aneuploids that may be naturally present in a population. Alternatively, FLC may act as a driving force, stimulating the formation of aneuploids from which a small proportion is selected for proliferation in the presence of the inhibitory concentration of the drug, similarly to the recently proposed mechanism in *C. albicans* and as hypothesized in studies that involved *C. neoformans* (8, 11). On the basis of the fact that *C. neoformans* is predominantly haploid, development of aneuploidy could proceed through formation of diploid cells, in analogy to the aneuploid formation seen on the basis of a tetraploid intermediate described for *C. albicans* (11). To test if FLC has an effect on DNA content of *C. neoformans* during initial exposure to the drug, cells treated with FLC were fixed, stained with propidium iodide (PI), and examined using fluorescence flow cytometry. Strikingly, treatment of cells with 32 μg/ml FLC at 24°C for 12 and 14 h resulted in levels of 20% and 88%, respectively, of the cell population with ploidy levels at or above 4N (Fig. 1). The significant increase of the cell number with a ploidy level at or above 4N between 12 and 14 h (Fig. 1) was not consistent from experiment to experiment, as maximum ploidy was present in some cases at around 9 h, with no significant further change at later time points (data not shown). The longest incubation time tested was 18 h, with results that did not show a further significant increase in the ploidy level (data not shown). The maximum ploidy level in a significant population of cells observed under any of the test conditions was approximately 4N, although a smaller fraction of cells with ploidy levels beyond 4N was also present at inhibitory concentrations of FLC (Fig. 1). The fraction of cells with increased DNA content was proportional to the concentration of FLC. Taken together, these data suggest that FLC treatment at inhibitory concentrations leads to an increase in ploidy beyond 2N.

### Treatment with FLC causes inhibition of budding.

We tested whether FLC treatment resulted in any of the following cellular defects that might individually or collectively result in increased DNA content: (i) inhibition of growth coupled with rereplication; (ii) a defect in cytokinesis; (iii) unequal levels of chromosomal segregation during mitosis.

**FIG 2:**
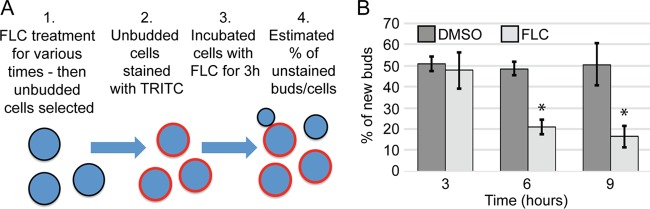
Treatment with FLC causes inhibition of budding. (A) Schematic showing the experimental procedure. After FLC treatment, the cell surface was stained with tetramethylrhodamine (TRITC). Stained cells were released into DMSO-containing control medium or 32 µg/ml FLC medium, and after 3 h the cells were imaged. The cells were counted using the bright-field channel, and the new buds (buds that were not stained) were counted using the rhodamine channel. (B) Analysis of new buds showed that after 6 and 9 h of FLC treatment, budding was significantly inhibited (*, *P* = 0.01).

The inhibitory effect of FLC, similarly to that of other azole antifungals, has been associated with the depletion of ergosterol from the plasma membrane (13). The minimum level of ergosterol needed to sustain cellular growth is not clear. The *C. neoformans* doubling time under unperturbed growth conditions in the laboratory is approximately 2 h (14). Therefore, during the initial period of FLC treatment, we expect the growth of cells to be relatively normal, as the depletion of ergosterol from the membranes may take more than a doubling time. Consistent with this prediction, we found that treatment of cells with 32 μg/ml FLC for 3 h resulted in a reduction of only ~20% in the level of new bud formation relative to the control results (Fig. 2). However, treatment for 6 h resulted in a more significant inhibition of budding (~50% reduction compared to the control; Fig. 2). These data suggest that the effect of FLC on growth is delayed, presumably due to the relatively low rate of exchange of ergosterol within the plasma membrane. If this were the case, we would expect that a further reduction in the exchange rate might lead to an even longer delay in the effect of FLC on bud initiation. Consistent with this possibility, the addition of actin depolymerizing agent latrunculin B (LatB) during the pretreatment of unbudded cells with FLC led to a further increase in the amount of time needed to significantly inhibit bud initiation; effective inhibition by FLC in combination with LatB was achieved by 14 h (see Fig. S1 in the supplemental material). Cells treated with LatB for 18 h initiated budding when released into drug-free media, which indicated that prolonged exposure to LatB did not have any significant permanent adverse effects on cell growth (Fig. S1). Taken together, these data suggest that FLC treatment leads to inhibition of bud initiation and subsequent growth. However, the effect is delayed. We speculate that levels of plasma membrane ergosterol reach a critical minimum after longer incubation with FLC, resulting in significant inhibition of the initiation of budding and further bud growth.

10.1128/mSphere.00205-17.1 FIG S1  Pretreatment of cells with FLC and actin inhibitor latrunculin B leads to delayed inhibition of budding through FLC treatment. A schematic of methodology explaining the experimental procedure is presented. Unbudded cells were selected and then treated either with LatB or with LatB plus FLC. Cells were then washed and released into DMSO or 32 µg/ml FLC, and budding was assessed. Treatment with LatB was used to assess the effect of the rate of ergosterol exchange on budding inhibition. Inhibition of budding was delayed during treatment with LatB plus FLC; unbudded cells that were treated with LatB plus FLC took 14 h for budding to be significantly inhibited. Download FIG S1, TIF file, 0.3 MB. Copyright © 2017 Altamirano et al. 2017 Altamirano et al. This content is distributed under the terms of the Creative Commons Attribution 4.0 International license.

### FLC treatment leads to a defect in cytokinesis.

**FIG 3:**
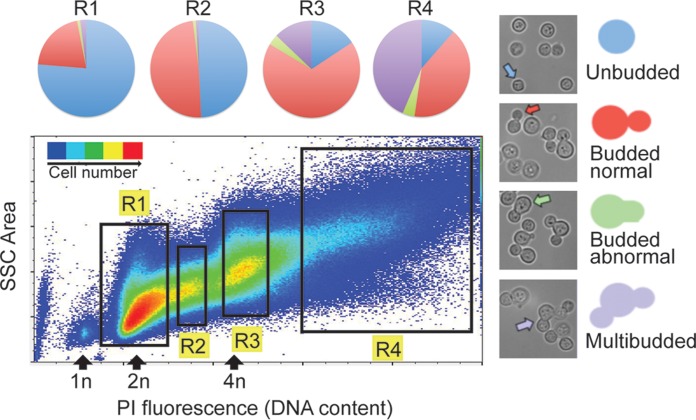
FLC treatment leads to a defect in cell separation. Cells were treated with 32 µg/ml FLC for 10 h and fixed, and the DNA was stained with PI. Subsequently, cells were fractionated using a fluorescence-activated cell sorter (FACS) instrument. Fractions of cells with increasing ploidy were collected, and morphology was scored under the microscope. The fractions of cells with highest ploidy level were enriched in multimeric cells. Multimeric cells (purple) either were trimeras, with two daughter cells formed from the mother cell, or contained an additional daughter (a granddaughter) grown from one of the daughters (purple arrow). SSC, side scatter.

Hypothetically, the fraction of cells with increased DNA content may represent a debilitating effect of FLC on cytokinesis. To test this hypothesis, we assessed the morphology of FLC-treated cells. To differentiate between cells with normal DNA content and those with increased DNA content, we fixed the cells, stained the DNA with PI, and subsequently fractionated the cells using a FACS (fluorescence-activated cell sorter) instrument. Fractions of cells with increased ploidy were collected, and the levels of morphology were scored under the microscope. Cells with a ploidy level close to 1N were predominantly unbudded or contained a single usually small bud (Fig. 3). Fractions with higher ploidy levels were enriched in cells characterized by the presence of two or more daughter cells (multimeric cells). These multimeric cells either were trimeras, with two daughter cells born from the mother cell, or contained an additional daughter (a granddaughter) born from one of the daughters (Fig. 3). A total of ~44% of the cells in the fraction with a ploidy level above 4N were multimeric cells. These results suggest that treatment with FLC leads to failure in cytokinesis. To rule out the possibility that multimeric cells result from cell fusion events, we mixed two strains, one that expressed histone H4 tagged with green fluorescent protein (GFP) and another that expressed H4 tagged with mCherry, and subjected the mixed-cell culture to FLC. While multimeric cells expressing either of the two fluorescent chimeras were frequent after longer incubations with FLC, we could not find multimeric cells that expressed both GFP and mCherry (data not shown). This is consistent with our hypothesis that multimeric cells result from cell division failure rather than from cell fusion events.

### FLC treatment leads to uncoupling of growth from the cell cycle.

**FIG 4:**
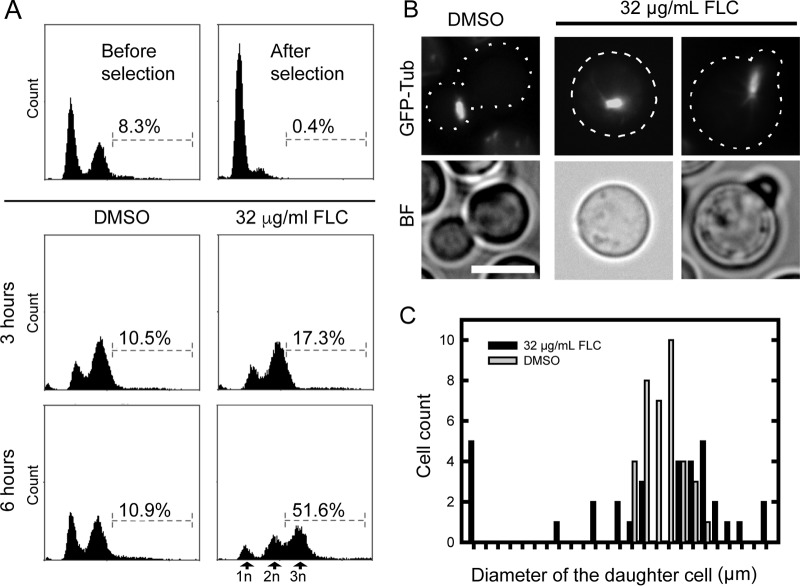
A delay or complete block in cell separation during FLC treatment may lead to an increase in DNA content in unseparated cells. (A) A population of preselected unbudded cells was incubated with 32 µg/ml FLC for 3 or 6 h (times when no multimeric cells were formed), and the DNA content was assessed using flow cytometry based on the PI staining. After 6 h of FLC treatment, ~50% of cells showed DNA content above 2N, with a predominant population (visible as a peak) that corresponded to cells with 3N DNA content. (B) FLC treatment results in an aberrant dynamics of the mitotic spindle. Localization of mitotic spindle in cells treated with 32 μg/ml FLC for 22 h was analyzed based on a strain that expressed GFP-tagged beta tubulin (GFP-Tub) (LK126). In a control sample (DMSO), the spindle was detected exclusively within daughter cells. In contrast, FLC-treated cells that had a detectable spindle either were unbudded or contained extremely small buds. BF, Brightfield. (C) The sizes of the daughter cells when the spindle was visible were relatively uniform for the control treatment (DMSO), while the FLC-treated cells exhibited a broader range of sizes of daughter cells when spindle was visible. Bar, 5 µm.

Interestingly, the fraction of cells with the highest ploidy also contained significant amounts of cells with a single bud (45%) and of unbudded cells (11%), suggesting that rereplication had taken place as a result of FLC treatment (Fig. 3). A delay or a complete block in cell separation during FLC treatment would potentially lead to an increase in DNA content in unseparated cells if the cell cycle were to continue and to result in subsequent rounds of replication. We tested this hypothesis further by subjecting a population of preselected unbudded cells to FLC treatment and assessing DNA content based on PI fluorescence using flow cytometry. Importantly, we examined cells after 3 and 6 h of treatment with 32 μg/ml FLC, which are times when no multimeric cells are detected in the population. After 6 h of FLC treatment, strikingly, ~50% of cells showed DNA content above 2N, with a predominant population (visible as a peak) that corresponded to cells with 3N DNA content (Fig. 4A). These results suggest that upon FLC treatment, cells exhibit a failure or a delay in cytokinesis with a concomitant new round of replication in the mother cell. An alternative, nonexclusive possibility is that FLC treatment might result in a failure to transition the chromatin to the daughter cell prior to metaphase, an event that normally occurs in *C. neoformans* (12). Hypothetically, failure to translocate the chromatin to the daughter cell might be followed by spindle formation and subsequent nuclear division in the mother cell leading to increased ploidy. To address this possibility, we analyzed the localization of the mitotic spindle in cells treated with 32 μg/ml FLC for 22 h, based on the use of a strain that expressed GFP-tagged β-tubulin. Consistent with previous studies, the spindle in the control sample was detected exclusively within daughter cells (Fig. 4B). In contrast, ~16% (*n* = 33) of FLC-treated cells that had a detectable spindle either were unbudded or contained extremely small buds and a spindle positioned within the mother cell (Fig. 4B). Consistently, we found that when the spindle was visible, the sizes of the daughter cells in the control were relatively uniform (*x̄* = 3.2; standard deviation [SD] = 0.31 µm). However, the FLC-treated cells exhibited a broader range of sizes of daughter cells when a spindle was detected (*x̄* = 3.5; SD = 0.84 μm) (Fig. 4C).

**FIG 5:**
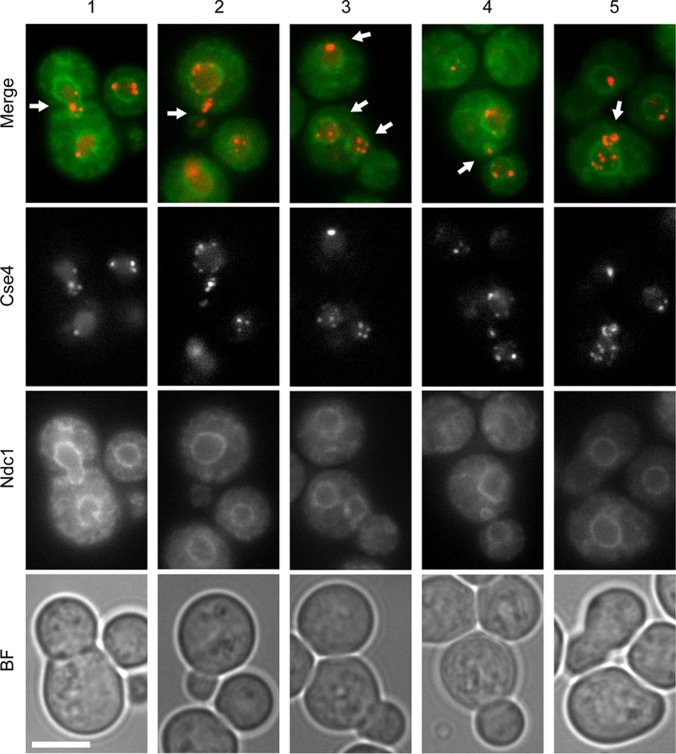
Analysis of centromere dynamics in FLC-treated cells. A strain that expressed a fluorescently tagged centromeric histone variant, mCherry-Cse4, and a component of the nuclear envelope, GFP-Ndc1 (CNV111), was subjected to treatment with 32 μg/ml FLC for 13 and 15 h prior to imaging. Panels 1 to 5 depict representative types of aberrations in Cse4 dynamics, including centromeres positioned at the mother-bud neck (panels 1 and 4, arrows), clustered centromeres in unbudded cells or cells with small daughters (panels 2 and 3), two nuclei present in one mother cell (panel 3), and centromeres that appear outside the nuclear area (panels 2 and 5). Bar, 5 µm.

To further assess the uncoupling of growth from the mitotic cycle, we monitored clustering of centromeres in FLC-treated cells. In *C. neoformans*, centromeres are not clustered in interphase cells or in cells with small buds, but they cluster in cells with medium-sized buds in preparation for mitosis (12). To visualize centromeres, we utilized a strain that expressed Cse4, a centromeric histone variant expressed from an endogenous promoter and tagged with mCherry, and Ndc1, a component of the nuclear envelope tagged with GFP. Strikingly, when cells were treated with 32 μg/ml FLC for 13 and 15 h, 50% and 35% of the unbudded cells, respectively, already showed the presence of clustered centromeres (Fig. 5, panel 3). In addition, 3% (13 h, *n* = 65) and 9% (15 h, *n* = 75) of budded cells contained 2 nuclei in the corresponding mother cell (Fig. 5, panel 3). Moreover, 3% (13 h, *n* = 65) and 4% (15 h, *n* = 75) of budded cells showed some centromeres in the bud neck area (Fig. 5, panels 1 and 4). Occasionally, we also observed cells with a portion of centromeres that localized outside the nuclear area marked by the Ndc1 (Fig. 5, panels 2 and 5). These data suggest that upon treatment with FLC, the mitotic cycle is less inhibited than growth and budding. This leads to clustering of centromeres, spindle assembly, and nuclear division in the mother cell, resulting in cells with increased DNA content and potentially aberrant chromosomal composition.

### Analysis of the mitotic defects resulting from FLC treatment.

**FIG 6:**
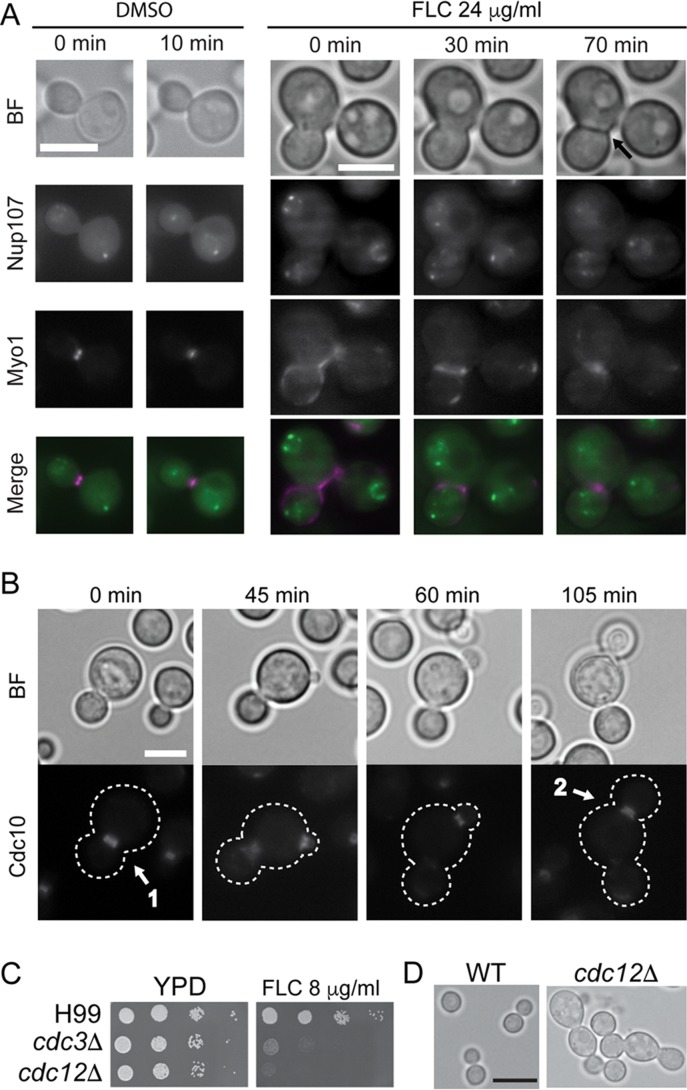
FLC does not significantly inhibit AMR assembly and constriction and septin localization. (A) To monitor constriction of the AMR, time-lapse microscopy was performed using a strain that expressed a component of the AMR, myosin heavy chain (Myo1), tagged with mCherry, and a nucleoporin (Nup107), tagged with GFP (to monitor the stage of mitosis; strain LC4). Cells from the control treatment (DMSO) had a constricted AMR. FLC-treated cells that formed multimeras also constricted the AMR, although the dynamics of the constriction differed significantly from those seen with untreated control cells. The arrow in the uppermost of the panels corresponding to the 70-min time point indicates a dark line that likely corresponds to a septum formed after the AMR had constricted. Bars, 5 µm. (B) Cells expressing septin Cdc10-mCherry (LK62) were pretreated with 24 μg/ml FLC for 6 h, and the localization of Cdc10-mCherry was analyzed by time-lapse microscopy while the cells were continually exposed to FLC (the times indicated represent the progression of the time lapse). Cdc10-mCherry formed a collar and a double ring between the mother and the first daughter (arrow 1) and formed another such ring when the second daughter was formed (arrow 2). (C and D) Cells deleted for Cdc3- or Cdc12-encoding genes (LK65, LK162) exhibited hypersensitivity to FLC (C), with a clear defect in cytokinesis at 24°C (D). Bars, 5 µm in panels A and B and 10 µm in panel D.

We investigated what specific defects accounted for the inability of cells to separate during FLC treatment. Defects in cell separation in basidiomycetous yeasts may result from a lack of actomyosin ring (AMR) constriction, from septum deposition failure, or from inadequate degradation of the primary septum toward the end of cytokinesis (15). To monitor constriction of the AMR, we performed time-lapse microscopy using a strain of *C. neoformans* that expressed a component of the AMR, myosin heavy chain (Myo1), tagged with mCherry and a nucleoporin (Nup107) tagged with GFP (to monitor the stage of mitosis). Cells with single daughters in both the control and FLC-treated samples exhibited normal AMR constriction (Fig. 6A and data not shown). However, FLC-treated cells with only a single daughter may be already partially resistant to FLC. Therefore, we focused on multimeric cells, as they represented the fraction of cells for which cell separation has failed. In all examined multimers (*n* = 7), the AMR constricted between the mother cell and the daughters (Fig. 6A; Fig. S2). Interestingly, the dynamics of the constriction differed significantly from that seen with the control cells. Specifically, while the constriction time seen with the dimethyl sulfoxide (DMSO)-treated control was estimated to be between 10 and 25 min (*n* = 9), the assessed constriction times in 5 multimeras that resulted from FLC treatment were 10, 20, 30, 35, and 50 min. Our results indicate that FLC does not inhibit AMR assembly but that FLC does cause a delay in AMR constriction.

10.1128/mSphere.00205-17.3 FIG S2  FLC does not significantly inhibit AMR assembly and constriction. (A) Time-lapse microscopy was performed with strain LC4 expressing GFP-Nup107 (to visualize the nuclear envelope; green) and mCherry-Myo1 (to visualize the AMR; magenta) after 6 h of preincubation with FLC. In the example shown, the AMR had constricted by 15 min of subsequent FLC treatment (white arrowhead), and a new bud had started to emerge by 135 min (white arrow) with no detachment of the first daughter cell. (B) A multimeric cell treated as described for panel A was imaged, and individual focal planes (Z-sections) are shown to illustrate a lack of cytoplasmic connection (based on the cytoplasmic signal of GFP-Nup107). In this cell, both of the daughter cells possessed the septa separating them from the mother cell. (C) The cell treated as described for panel B was imaged at an earlier time point when the second daughter had not developed a septum and its cytoplasm had therefore not yet separated from the mother. To assess separation of the cytoplasmic signal, pixel brightness along a line perpendicular to the mother-daughter axis was plotted for both daughters as shown. While the data from the second daughter (top graph) show a steady increase of fluorescence along the line drawn, the data from the first daughter show diminishment of the fluorescence in the area corresponding to the mother-bud neck, suggesting the presence of discontinuous cytoplasm between the two cells. Bars, 5 µm. Download FIG S2, TIF file, 2 MB. Copyright © 2017 Altamirano et al. 2017 Altamirano et al. This content is distributed under the terms of the Creative Commons Attribution 4.0 International license.

Septin proteins in *C. neoformans* are essential for cytokinesis under stress conditions (16). Therefore, incomplete cytokinesis upon FLC treatment may stem from the inability of cells to assemble a robust septin complex at the site of cytokinesis. However, we did not observe marked defects in septin localization or organization upon FLC treatment (Fig. 6B). The septins (Cdc3 and Cdc12) are essential for the assembly of the septin complex at the mother-bud neck, and yet *cdc3*Δ or *cdc12*Δ mutant strains exhibit largely normal cytokinesis under stress-free conditions (24°C), albeit having partially abnormal primary septa (16). Strikingly, *cdc3*Δ and *cdc12*Δ strains exhibited hypersensitivity to FLC with a clear defect in cytokinesis at 24°C (Fig. 6C and D). Synthetic interaction between septin deletions and FLC further suggests that FLC-mediated inhibition of cytokinesis is not directly related to septin function.

Calcineurin high temperature suppressor 1, Cts1, has been implicated in the cell separation pathway in *C. neoformans*. Cts1 is thought to contribute to primary septum formation (17). GFP-Cts1 localizes to the mother-bud neck following the constriction of the AMR during cytokinesis (18). FLC treatment did not affect the localization and dynamics of GFP-Cts1, a finding consistent with the septum being formed during FLC treatment (Fig. S3A and B).

10.1128/mSphere.00205-17.2 FIG S3  Analysis of the dynamics of Cts1 and deposition of the chitin in FLC-treated cells. Panels A and B depict cells expressing GFP-Cts1 and mCherry-Myo1 (LK274) treated with 24 µg/ml FLC and imaged using time-lapse microscopy. (A) Constriction of the actomyosin and Cts1 rings occurred within 24 min in the bud neck between the first daughter and mother cells (arrows). The first daughter cell failed to separate, while a new bud emerged after 184 min (BF panel, arrow). Microtubules resembling structures of Cts1 were seen (arrowhead). (B) Formation of the Cts1 ring follows that of the actomyosin ring. Constriction of the AMR takes place at 50 min, while the constriction of the Cts1 ring follows 10 min later. (C) Cells treated with FLC for 9 h were stained with calcofluor white and show the presence of chitin at the mother-bud neck of multimeric cells. Bars, 5 µm. Download FIG S3, TIF file, 2.9 MB. Copyright © 2017 Altamirano et al. 2017 Altamirano et al. This content is distributed under the terms of the Creative Commons Attribution 4.0 International license.

To assess septum formation, we stained FLC-treated cells with calcofluor white to visualize chitin deposition. We noted that in the control cells, the calcofluor fluorescence was visible at the mother-bud neck in small, medium-sized, and large budded cells (data not shown). This implies that chitin may be deposited at the bud neck prior to mitosis in addition to the occurrence of the primary septum formation, similarly to *S. cerevisiae* (19). Importantly, we did not find a significant diminishment of the calcofluor fluorescence at the mother-bud neck of cells treated with FLC, including the multimeric cells, indicating that FLC does not prevent septum formation (Fig. S3C). In support of this conclusion, we observed a dark line at the bud neck between the mother and the daughters in the trimeric cells in imaging in the bright field (Fig. 6; Fig. S2). In addition, cytoplasm was discontinuous between the mother and the daughter cells in multimeric cells when the dark line became visible at the mother-bud neck (Fig. S2B and C).

Taken together, our data suggest that FLC inhibits final separation of cells undergoing cytokinesis but that this defect does not result from a lack of AMR constriction or septum formation or a lack of Cts1 localization and constriction. Instead, the defect is a consequence of the failure of the final degradation of the primary septum.

### Chromosomal loss is not the predominant mechanism responsible for generating FLC-resistant cells.

Harrison et al. have proposed that a diploid *C. albicans* cell becomes aneuploid under conditions of FLC treatment through generation of an intermediate tetraploid stage (11). Extrapolating from this scenario, we could envision a haploid *C. neoformans* cell becoming a diploid cell in the presence of FLC and that the diploid cell would subsequently become an aneuploid cell through concerted losses of chromosomes. Hypothetically, a diploid strain of *C. neoformans* would possess a much higher potential to become aneuploid as a consequence of FLC-triggered chromosomal loss; therefore, the likelihood of a diploid strain becoming resistant to FLC should be higher. To test this hypothesis, we artificially generated a diploid *C. neoformans* strain and compared it to two parent haploid strains with respect to early response to FLC and the ability to form FLC-resistant colonies. Grown on 32 μg/ml of FLC media, the diploid produced visible colonies after 72 h, whereas the two haploid parent strains produced no visible colonies at that time (data not shown). However, diploid cells grown in the presence of FLC reached an octoploid (8N) state after 14 h in a manner analogous to the DNA increase observed in the haploid strain (Fig. S4). Morphological examination of the diploid cells grown in FLC showed multibudded cells, indicative of cytokinesis failure similar to that observed in haploid cells (data not shown). These findings imply that diploid *C. neoformans* cells, while potentially less sensitive to FLC than haploids, in a manner analogous to that seen with the haploid cells, fail to undergo cytokinesis and double the ploidy level upon FLC treatment. This suggests that chromosomal loss is not sufficient and is not the predominant mechanism of aneuploidy formation in response to FLC. Furthermore, these data indicate that a diploid state is not sufficient to render cells resistant to a concentration of FLC that is inhibitory to the isogenic haploid.

10.1128/mSphere.00205-17.4 FIG S4  FLC treatment results in an increase in ploidy in a significant fraction of diploid cells. Two haploid strains (parent 1 [LK315] and parent 2 [CNV121]) and two diploids, namely, a diploid derived from the two haploids (DSA3) and a reference diploid (Bt163), were treated with 32 µg/ml FLC, fixed, stained with PI, and passed through a fluorescence flow cytometer to assess ploidy. Both diploids underwent increase in ploidy analogous to results seen with the haploid strains (parents of derived diploid and wild-type strains), suggesting that 32 µg/ml of FLC imposes similar inhibitory effects on haploids and diploids. Therefore, diploids are not significantly resistant to FLC compared to the isogenic haploids. Download FIG S4, TIF file, 2.4 MB. Copyright © 2017 Altamirano et al. 2017 Altamirano et al. This content is distributed under the terms of the Creative Commons Attribution 4.0 International license.

### FLC treatment leads to unequal distributions of chromatin between the mother cells and the daughter cells.

**FIG 7:**
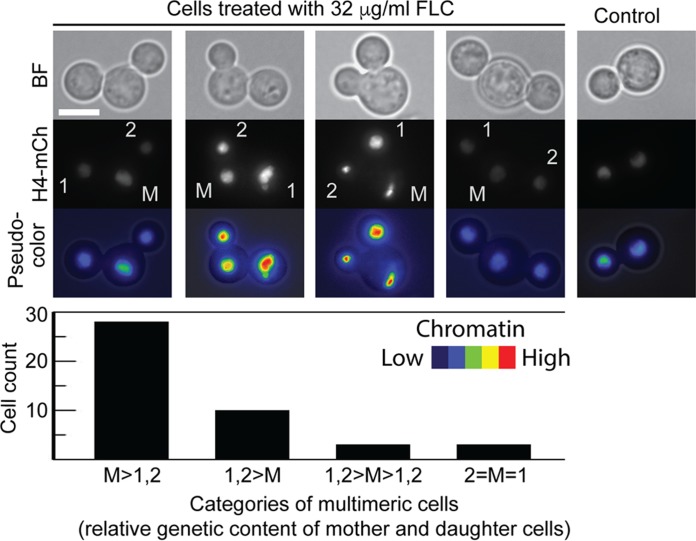
FLC treatment leads to unequal distributions of chromatin between the mother and the daughter cells. Cells expressing histone H4-mCherry (H4-mCh) from the endogenous promoter (CNV121) were treated with 32 μg/ml FLC for 12 h and imaged using Z-section fluorescence microscopy. A total of 44 multimeric cells were evaluated for the H4-mCherry signal in each of the cells constituting a trimera (M, mother; 1, first daughter; 2, second daughter). Cells were grouped depending on the relative amount of H4-mCherry fluorescence in each of the three cells as described in detail in Materials and Methods. The cells belonging to each category were counted as indicated. The meaning of each category is as follows: M>1,2, more signal in the mother than in either of the daughter cells; 1,2>M, more signal in either of the daughter cells than in the mother; 1,2>M>1,2, one daughter with more and one with less signal than the mother; 2=M=1, all three cells with equal levels of signal. Bar, 5 µm.

We utilized a strain of *C. neoformans* that expressed histone H4 tagged with mCherry as a proxy to estimate the levels of distribution of chromatin during mitosis in cells treated with FLC. The strain expressing H4-mCherry exhibited sensitivity to FLC similar to that exhibited by the wild-type strain, indicating that introducing mCherry at the C terminus of the histone H4 did not change the way that FLC affected cell division (data not shown). In DMSO-treated control cells, the chromatin was equally distributed between the daughter and the mother cell, as the ratio of the levels of H4-mCherry fluorescence between the daughter and mother cells was nearly 1 (*x̄* = 1.13; SD = 0.28; *n* = 19). In contrast, some multimeric cells that were treated with FLC exhibited markedly unequal distributions of the H4-mCherry signal between the mother and the daughter cells (Fig. 7). Interestingly, there was no consistency as to whether the greatest amount of H4-mCherry signal was in the mother or in any of the daughters, although the predominant fraction of multimeras contained more chromatin in the mother than in either of the daughters (Fig. 7). These data suggest that FLC treatment results in aberrant chromosomal distribution leading to the presence of cells with relatively higher chromatin content.

### FLC-treated cells that are enlarged and/or fail to separate are less sensitive to FLC.

**FIG 8:**
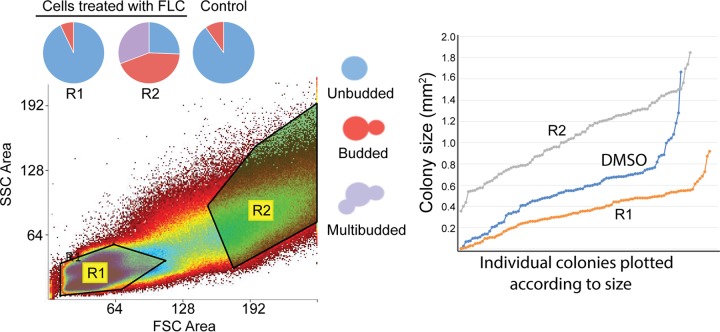
FLC-treated cells that are enlarged and/or fail to separate are less sensitive to FLC. Cells were treated with 32 μg/ml FLC for 14 h. Two separate fractions (fractions R1 and R2) were obtained based on size and complexity of morphology using FACS analysis. The morphology of each fraction was assessed. Fraction R1 consisted of predominantly unbudded cells with a smaller fraction of budded cells. Fraction R2 consisted of approximately equal amounts of unbudded cells, budded cells, and multibudded cells. Cells were then plated on media containing 32 μg/ml FLC, and the areas of the colonies were measured after 3 days. Fraction R1 did not produce as many large colonies as fraction R2, suggesting that the enlarged and/or multibudded cells were relatively less sensitive to FLC.

Our data suggest that FLC has a pleiotropic effect on cell growth, including inhibition of budding and cell separation and missegregation of chromatin during mitosis. Collectively, these effects result in cells that are increased in size, have higher DNA content, and are multibudded. Indeed, on the basis of the fluorescence microscopy profile of the cells treated with 32 μg/ml FLC for 14 h, we found a significant correlation between cell size and complexity and the DNA content (Fig. 1 and data not shown). We hypothesized that enlarged cells are less sensitive to FLC. To test this, cells that had been treated with 32 μg/ml FLC for 14 h were fractionated using a FACS instrument on the basis of their size and the complexity of their morphology. Two fractions were collected, one with relatively small cells that either were unbudded or contained a single bud and a second that contained unbudded cells or budded cells that were enlarged or multimeric (Fig. 8). A third (control) sample consisted of cells that were not fractionated and yet were processed the same way, including passing through the FACS instrument. All three samples were plated on media containing 32 μg/ml of FLC and were incubated at 24°C. Significantly, the fraction of cells containing multimeras exhibited higher resistance to FLC than the fraction of “small” cells (Fig. 8). Cells that were not subjected to fractionation exhibited an ability to grow in the presence of FLC that was intermediate between those of the other two samples. These findings indicate that cells that are enlarged and/or have changed morphologically upon treatment with FLC possess a higher potential to grow in the presence of the drug.

These findings (Fig. 8), coupled with our initial FACS data showing the morphology of cells with increased DNA content (Fig. 3), highlight the role of multimeric cells as a factor contributing to the increase in ploidy and subsequent survival in the presence of FLC. Additionally, the findings emphasize that enlarged, unbudded cells are potentially another factor contributing to survival. In order to better understand the role of enlarged morphology, cells were streaked in the middle of a plate containing 24 µg/ml FLC medium. After 9 h, before the emergence of multimeras, 20 enlarged unbudded or budded cells were picked using a micromanipulator and placed on the same plate in known spots. A total of 20 cells of normal size were also placed as controls. Six of the 20 enlarged cells progressed into colonies, while 0 of 20 of the control cells of normal size grew. These data are not surprising; the cells that showed an increase in survival were able to grow or enlarge during the initial insult of FLC, whereas the nonsurvivors were unable to handle the stress imposed by FLC. The microcolonies formed by the six enlarged cells at 24 h were dissected, and the morphology and subsequent mitotic divisions were monitored for the succeeding 24 h. Cells that were already multimeras by 24 h exhibited the greatest chance of survival in the presence of FLC (formed microcolonies); ~89% of multimeras underwent further growth (Fig. S5). In contrast, ~50% of budded cells and ~30% of unbudded cells formed microcolonies. The heterogeneity of the population with respect to FLC resistance is intriguing. It will be of interest to further explore the basis and the importance of the heterogeneity of the initial response to FLC.

10.1128/mSphere.00205-17.5 FIG S5  Microdissection of colonies grown on FLC media. Slightly enlarged budded or unbudded cells were placed on specific regions of a 32 µg/ml FLC plate using a micromanipulator. After 24 h, 6 microcolonies derived from the enlarged cells were dissected, separating each of the cells within a colony (2 representative dissections are shown). The morphology of each cell at that point was assessed. Cells were then imaged at the 36-h and 48-h time points, and the number of resulting microcolonies was scored. Quantification of the 6 microcolonies at 48 h with respect to the initial morphologies of the cells at 24 h (after dissection) was performed as shown in the graph. Multimeric cells developed into significantly more microcolonies than single-budded or unbudded cells. Download FIG S5, TIF file, 2.9 MB. Copyright © 2017 Altamirano et al. 2017 Altamirano et al. This content is distributed under the terms of the Creative Commons Attribution 4.0 International license.

## DISCUSSION

Our data suggest that FLC treatment results in an increase in the DNA content in a significant fraction of cells but that the effect is delayed. What is most likely to account for the delay is the rate at which ergosterol is depleted in individual cells; however, other reasons cannot be excluded. For instance, relatively slow accumulation of a toxic by-product of the FLC-mediated inhibition of ergosterol synthesis may also be responsible for the delay (10). Significant inhibition of budding of cells treated with 32 μg/ml of FLC occurs at 6 h, a time when no significant increase of DNA content is yet observed in a heterogeneous population. However, it required only ~3 h for a significant population of cells corresponding to 3N to appear when the initial population was composed of unbudded cells. Thus, an initial increase in DNA content is likely to occur due to a lack of cell separation coupled with subsequent replication in the mother cell.

FLC inhibits cytokinesis in *C. albicans*, but the actual mechanism of cytokinesis failure has not been explored (11). While we also find that FLC causes inhibition of cell separation in *C. neoformans*, two main differences from *C. albicans* are apparent. First, the *C. albicans* multimeric cells resulting from FLC treatment consist of the mother cell and the daughters that remain connected via common cytoplasm (11). Harrison et al. speculate that the cytoplasmic signaling connection between individual cells within the multimera is important for the formation of the tetraploid intermediate (11). In contrast, we observed that multimeric *C. neoformans* cells are formed on the basis of a failure of the final cell separation process despite septa being formed. Therefore, the mother cell and the first daughter do not share cytoplasm when the second daughter is formed, which is consistent with images of multimeras with discontinuous fluorescent cytoplasmic signal between the attached cells (see Fig. S2 in the supplemental material). A second clear distinction is that the multimeric cells in *C. albicans* are chains of cells resulting from growth of the second daughter cell (a granddaughter) out of the first daughter cell, while in *C. neoformans*, both daughter cells grow consecutively from a single mother. We speculate that retention of the cytoplasmic connection in *C. albicans* and a lack thereof in *C. neoformans* account for this difference. Specifically, in *C. albicans*, cell cycle signaling that normally triggers the next round of budding in the mother is acting within the first daughter, which results in a granddaughter. In *C. neoformans*, the mother cell of the multimera is isolated from the unattached daughter and the signaling triggers formation of a second bud within the mother. Indeed, we observed multimeras with two daughters attached to the mother and a single granddaughter, indicating a later time at which one of the daughters is “ready” to initiate budding (Fig. 3, purple arrow). It is interesting that, in contrast to *C. albicans*, FLC treatment does not prevent septation in *C. neoformans*. This may indicate that either the physical processes of AMR constriction and/or septum formation differ in these species or, alternatively, the differences in the signaling that triggers these events account for various effects of FLC in these species. Given that both species produce aneuploid progeny presumably based on multimeric cells, the issue remains of whether aneuploidy formation proceeds through distinct mechanisms in these unrelated yeasts. Alternatively, at least in *C. neoformans*, these multimeric cells may not constitute a critical prerequisite to truly resistant aneuploids but rather a by-product of FLC inhibition that nonetheless increases the survival of cells in the presence of the drug. Addressing these issues will require further investigation.

Fernández et al. demonstrated that cholesterol, the mammalian equivalent of ergosterol, was required for cytokinesis in a human HL-60 cell line and that sustained cholesterol starvation led to the formation of polyploid, multinucleated cells with mitotic aberrations (20). They hypothesized that the cell cycle perturbations and polyploidization observed in cholesterol-deficient cells are due to reduced Cdk1 activity. Furthermore, affected cells were able to partially traverse mitosis and to rereplicate DNA, which led to polyploidy (20). Thus, it is likely that the cell separation defect that we observed in *C. neoformans* was due to depletion of ergosterol. Our data suggest that FLC prevents cell separation in *C. neoformans* most likely via inhibition of the final degradation of the primary septum, as the primary septum was formed and the two other main events of cytokinesis, AMR constriction and septin assembly, were largely not affected. Surprisingly, none of the endochitinases encoded by the *C. neoformans* genome are necessary for the final cell separation (21, 22). *C. neoformans* may not have an enzyme that specifically hydrolyzes chitosan, a constituent of the primary septum. Therefore, daughter cell separation may proceed based on the increased flexibility and solubility of the chitosan (21). It is plausible that FLC disrupts the relative content of chitosan, leading to defects in cell separation. Alternatively, a delay in AMR constriction or other indirect effects of FLC may cause an interruption of a conserved RAM (regulation of Ace2 and morphogenesis) pathway that signals final cell separation (23).

**FIG 9:**
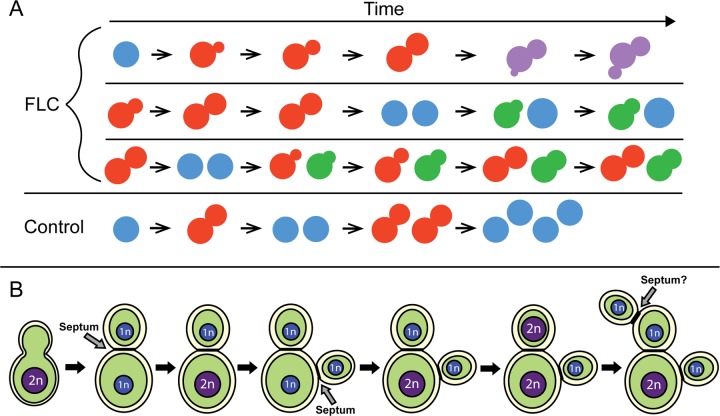
(A) Model illustrating the effects of FLC on the cell growth, cell cycle progression, and cytokinesis that result in cells with increased ploidy, including single cells, multimeric cells, and cells with abnormal buds. According to this model, the resulting morphological defect for a given cell depends on the cell cycle stage during which the cell was exposed to FLC initially. (B) Model depicting the progression of the events that lead to formation of a multimeric cell during exposure to FLC. In this model, a multimera with equally distributed chromatin levels may transition to a stage during which more chromatin is present in the mother cell than in the daughters. While this seems to be the most prevalent type of multimeric cell, cells with other chromatin distribution patterns and mother cells with two nuclei were also found (Fig. 5 and 7).

Several abnormal morphologies resulting from FLC treatment reflect its diverse effects on cell physiology. We propose that the variability in morphological defects results from the heterogeneity that exists in the population of cells that is initially exposed to the drug. In its simplest form, the heterogeneity may reflect cells that are at various stages of the cell cycle when they are initially exposed to the drug (Fig. 9A). FLC treatment leads to a gradual depletion of ergosterol and an accumulation of toxic metabolic products. Consequently, cells are affected in various ways depending on when ergosterol levels reach the critical minimum with respect to the stage of the cell cycle (Fig. 9A). Interestingly, populations of cells exposed to FLC exhibit variability in the initial response to the drug (Fig. S5). It will be of interest to investigate the basis for this heterogeneous response.

Cells that fail to separate, and yet undergo DNA replication and subsequent initiation of budding and then proceed through additional rounds of mitosis (multimeric cells), most likely constitute a major group that accounts for the increased DNA content as detected via PI staining and flow cytometry (Fig. 9B). The fact that the mother cells undergo replication despite the failure in daughter cell separation is consistent with previous findings demonstrating that the timing of replication in *C. neoformans* is flexible with respect to the timing of the bud initiation (14). Therefore, we speculate that inhibition of cell separation by FLC treatment may be sufficient to result in the next round of replication in those mother cells. Consistent with the contribution of multimeras to the fraction of cells with increased ploidy as detected by flow cytometry, we found multimeric cells with increased and unequally distributed chromatin levels as judged by the fluorescence of H4-mCherry. Currently, the mechanism through which FLC treatment leads to unequal distributions of histone H4 is unclear. This defect is likely associated with missegregation of chromosomes, as we also observed aberrant segregation of centromeres between mother and daughter cells (Fig. 5). In *S. cerevisiae*, increases in ploidy are associated with chromosomal instability (24, 25). Hypothetically, missegregation of chromosomes may be a direct mechanism through which aneuploids are derived. A nonexclusive alternative is that cells with increased ploidy may undergo stepwise chromosomal loss, leading to aneuploidy. FLC causes inhibition of budding, which, when coupled with subsequent replication, would lead to an increase in DNA content. In addition, we observed premature mitosis occurring within mother cells, which is consistent with the presence of unbudded cells in the fraction with increased ploidy. Thus, FLC treatment results in an increase in DNA content by affecting cellular growth and division via multiple mechanisms. The central issue is whether the cells with increased DNA content that are formed in the presence of FLC give rise to a population of aneuploids that increases the chance of producing resistant populations. Our results, which show that cells with increased size and aberrant morphology grow better in the presence of FLC, are consistent with this possibility.

While increased ploidy due to FLC treatment may not be sufficient to generate truly resistant aneuploids in the host, the presence of cells with increased DNA content may increase the chance for survival in the presence of the drug and may hypothetically lead to more persistent infections. Recent studies showed a *C. neoformans* morphological variant of vastly increased ploidy and size called the Titan cell (26). Titan cells are found during infection and are more resistant to stress and FLC. Additionally, they produce populations of more-resistant aneuploids (27). FLC treatment is not sufficient to produce Titan cells, and the mechanism through which FLC increases DNA content *in vitro* appears different from that associated with the ploidy increase seen in Titan cells during infection. However, FLC-treated mice infected with *C. albicans* show morphologically changed yeast cells that likely stem from an inability to undergo cytokinesis (11). Isolates of *C. neoformans* obtained from clinical samples exhibited significant variation in susceptibility to FLC, and resistant clones with chromosomal disomy have been detected in brains of mice treated with FLC (28, 29). Thus, our *in vitro* data suggest that during infection with *C. neoformans*, treatment with FLC may lead to an increase in DNA content in yeast cells through pleiotropic effects on cell division. Cells with increased DNA content would support microevolution of populations with an augmented potential to survive in the host environment.

As discussed by Cheong and McCormack, several studies demonstrated conflicting results regarding the correlation between the MIC values for FLC seen *in vitro* and the clinical outcomes (30). The authors demonstrated that 30% of patients with cryptococcosis who have never been exposed to FLC showed evidence of reduced susceptibility to this antifungal (30). Desnos-Ollivier et al. have recovered genetically related haploid and diploid strains from the same patients and demonstrated through experimental infections and quantitative PCR that ploidy changes can result from endoreplication and that switching between haploid and diploid states can occur, consistent with microevolution within the host (31). Therefore, it is plausible that stress conditions in the host stimulate an increase in DNA content through inhibition of cellular division that is similar to the *in vitro* effects of FLC described here. Several genes have been described in *S. cerevisiae* that are essential for viability of polyploid cells (25). It would be of interest to test if homologues of these genes in *C. neoformans* are essential for viability of FLC-derived polyploids and formation of FLC-resistant populations.

## MATERIALS AND METHODS

### Growth conditions.

Strains used in this study are listed in Table 1. Unless otherwise stated, cells were grown in liquid yeast extract-peptone-dextrose (YPD) media overnight at 24°C and were refreshed the next day to an optical density at 600 nm (OD_600_) of 0.2 before treatment. For FLC-treated cultures, 50 mg/ml FLC stock solution (Sigma, St. Louis, MO, or Alfa Aesar, Haverhill, MA) was prepared in DMSO. Spot assays were performed using a 10-fold serial dilution starting with 10,000 cells per 5 µl and ending with 10 cells per 5 µl. Cells were spotted on semisolid YPD media or YPD media containing indicated concentrations of FLC, incubated at room temperature, and imaged after 3 days.

**TABLE 1:** List of strains

Strain	Genotype	Reference or source
H99	α WT^a^	36
Bt163	Diploid; environmental isolate	37
CNV111	**a** *GFP-NDC1*::*NAT* + *mCherry-CSE4*::*NEO*	12
CNV121	α *H4-mCherry*::*NEO*	33
DSA3	**a**/α *H4-mCherry*::*NEO*/*H4 GFP-NUP107*::*NAT*/*NUP107*	This study
LC4	**a** *GFP-NUP1*::*NAT mCherry-MYO1*::*HYG*	This study
LK62	**a** *CDC10-mCherry*::*NEO*	16
LK65	α *CDC3*::*NAT*	16
LK162	α *CDC12*::*NEO*	16
LK274	**a** *GFP-CTS1*::*NAT mCherry-MYO1*::*HYG*	18
LK126	α *GFP-TUB*::*NAT*	This study
LK315	**a** *GFP-NUP107*::*NAT*	This study

a WT, wild type.

### Strain constructions.

All transformations were performed using biolistics transformation (32). The diploid (DSA3) was generated by mating a strain expressing nucleoporin Nup107 tagged with GFP, LK315 (constructed as described earlier based on plasmid pCN19 [16]), with a strain expressing an endogenous histone H4 tagged with mCherry, CNV121 (33). The two strains were mixed on the mating MS agar medium, and after 2 days, cells were plated on double-selection (nourseothricin [NAT] and hygromycin B [HYG]) media, from which diploids were recovered. Diploid generation was confirmed using fluorescence microscopy and flow cytometry. Strain LC4 was generated by transforming LK315 with plasmid LKB77 (18). Strain LK126, expressing GFP-tagged beta tubulin (CNAG_01840), was generated by transforming strain H99 with plasmid LKB37 (a pCN19-based plasmid expressing GFP-tagged beta tubulin from a constitutive histone H3 promoter). Strain LK65 (*cdc3*Δ) is identical to the previously published LK64 strain (16).

### Flow cytometry.

Cells were harvested before exceeding an OD_600_ of ~0.8, spun down, washed with sterile water, suspended in 100 µl distilled water, and fixed with 70% ethyl alcohol (EtOH) (in a dropwise manner with vortex mixing). Cells were then incubated at 24°C for 1 h and transferred to 4°C overnight. The next day, cells were washed with RNase A buffer (0.2 M Tris [pH 7.5], 20 mM EDTA), suspended in 100 µl of RNase A buffer with 1 µl RNase A (from 10 mg/ml stock), and incubated for 4 h at 37°C. Cells were then washed twice with 1 ml phosphate-buffered saline (PBS), suspended in 900 µl of PBS, and incubated at 4°C overnight. Cells were stained with propidium iodide (PI) by addition of 100 µl of 0.005 µg/ml PI stock and incubated in the dark for 30 min. Immediately before analysis, cells were sonicated at an amplitude of 20% for 5 s to avoid clumping.

For ploidy analysis, PI fluorescence data were collected from 10,000 cells using FL3 (488-nm-wavelength laser) on a BD Accuri C6 flow cytometer. To assess the morphology of cells according to ploidy levels, cell sorting was performed using a Bio-Rad S3E cell sorter. At least 500,000 cells were sorted into each fraction based on PI fluorescence, and the morphology of cells was assessed using the following categories: unbudded, normally budded, abnormally budded (wide neck), and multimera.

To assess the FLC susceptibility of cell populations according to cell size, cell sorting based on cell size and complexity was performed using a Bio-Rad S3E cell sorter. Cells from each fraction (R1, R2, and DMSO control) were scored based on morphology using the following categories: unbudded, budded, and multimera. Cells from each fraction were then plated on semisolid YPD media containing 32 µg FLC and grown at 24°C for 6 days. After 6 days, plates were imaged and areas of random sets of 100 colonies were measured using ImageJ (34).

For analysis of rereplication, unbudded cells were selected via a modified centrifugation method based on a procedure described by Ayscough et al. (35). Cells were treated for 3 h with 32 µg/ml FLC or an equivalent level of DMSO and were then pelleted and suspended in 50% sorbitol (1 M) and 50% YPD media. Cells were spun at 2,000 rpm for 5 min. The supernatant was transferred to a new tube and spun at 1,500 rpm for 5 min. The supernatant was then transferred to a new tube again and spun at 4,000 rpm for 10 min. The morphology of the pelleted cells was then assessed under a microscope to confirm that the majority of the population was unbudded. Cells were released into DMSO or 32 µg/ml FLC. The morphology was checked at various time points to confirm that no multimeric cells were present, and the cells were fixed and stained with PI for ploidy assessment using flow cytometry.

### Microscopy.

Bright-field and fluorescence images were captured using a 100× objective with a Zeiss Axiovert 200 inverted microscope (Carl Zeiss, Inc., Thornwood, NY) interfaced with AxioVision Rel 4.8 software (Carl Zeiss, Inc., Thornwood, NY). Micromanipulation was performed using a SporePlay dissection microscope (Singer Instruments, United Kingdom). Unless otherwise stated, images were processed in Adobe Photoshop (Adobe Systems, San Jose, CA). Zen blue (Carl Zeiss, Inc., Thornwood, NY) was used to measure the diameter of the bud of mitotic spindle-containing cells. ImageJ (34) was used to measure H4-mCherry nuclear fluorescence. This was done by flattening the Z-sections to project the maximum intensities from each section and outlining the fluorescence of the nucleus. To account for DNA compaction, the pixel value of the outlined area was multiplied by the area. To establish a criterion on which to base the grouping of cells according to their relative levels of H4-mCherry fluorescence, we first measured variations of the fluorescent signal in control cells (not exposed to FLC). Specifically, we found that the ratio of the fluorescent signals of the daughter and mother cell ranged between 0.7 and 1.6. On the basis of this variation, we decided to use a ratio of less than 0.7 and a ratio of more than 1.6 to define lower and higher levels of chromatin in the daughter cell than in the mother cell, respectively.

For time-lapse analysis of components of cytokinesis, cells were pretreated with 24 µg/ml FLC in YPD or yeast nitrogen base (YNB) medium with 2% glucose at 24°C for ~6 h. Subsequently, cells were transferred to YNB–2% glucose medium containing 24 µg/ml FLC (300 µl of 1.00 × 10^6^ cells/ml·cell suspension) and were placed in a chamber of a borosilicate 8-chamber slide (Bio-Tek, Winooski, VT). At each time point, images of 5 Z-sections spaced 1.20 µm apart were taken.

To visualize chitin, calcofluor white staining was performed. H99 cells were grown in YPD media and treated with either 24 µg/ml or 32 µg/ml FLC for 9 h. Cells were then harvested, washed with YNB media, and fixed with 3.7% formaldehyde for 1 h while the culture was aerated. Cells were washed with PBS and permeabilized with 1% Triton X (Sigma-Aldrich, St. Louis, MO) for 10 min. Finally, cells were incubated for 30 min after the addition of 1 µg/ml of calcofluor white (Sigma-Aldrich, St. Louis, MO), washed, resuspended in YNB media, and visualized with a Zeiss Axiovert 200 inverted microscope.

For evaluation of budding, cell surface was biotinylated using EZ-Link sulfo-NHS-LC-Biotin (ThermoScientific) and stained with ExtraAvidin tetramethylrhodamine (TRITC) (Sigma, St. Louis, MO). Cells were washed three times with PBS and suspended to a density of 5 × 10^7^ cells/ml. Subsequently, 4 mg/ml of sulfo-NHS-LC-Biotin reagent was added. Cells were incubated at 24°C for 30 min. Cells were washed three times with YPD media. Biotinylated cells were released into 2 ml of YPD media with DMSO or 32 µg/ml FLC, and after 3 h, the cells were washed three times with PBS and incubated in the dark with TRITC (1:200) for 10 min. Cells were washed three times with PBS and imaged. The cells were counted using the bright field. Then, the new buds (buds that were not stained) were counted using the rhodamine channel.

To assess the effect of the ergosterol exchange rate on budding inhibition, unbudded cells were selected via centrifugation (35). Cells were then incubated with either 100 µm LatB (Enzo Life Sciences, Inc., Farmingdale, NY) or 100 µm LatB–32 µg/ml FLC for various time periods. Cells were then washed and incubated for an additional 3 h in DMSO or 32 µg/ml FLC. The percentage of budded cells was then estimated based on bright-field microscopy.
