# Supplementary figures and images for: Fluconazole-Induced Ploidy Change in Cryptococcus neoformans Results from the Uncoupling of Cell Growth and Nuclear Division
Source: mSphere. 2017 Jun 14;2(3):e00205-17. doi: 10.1128/mSphere.00205-17 (PMC5471349; doi:10.1128/mSphere.00205-17)

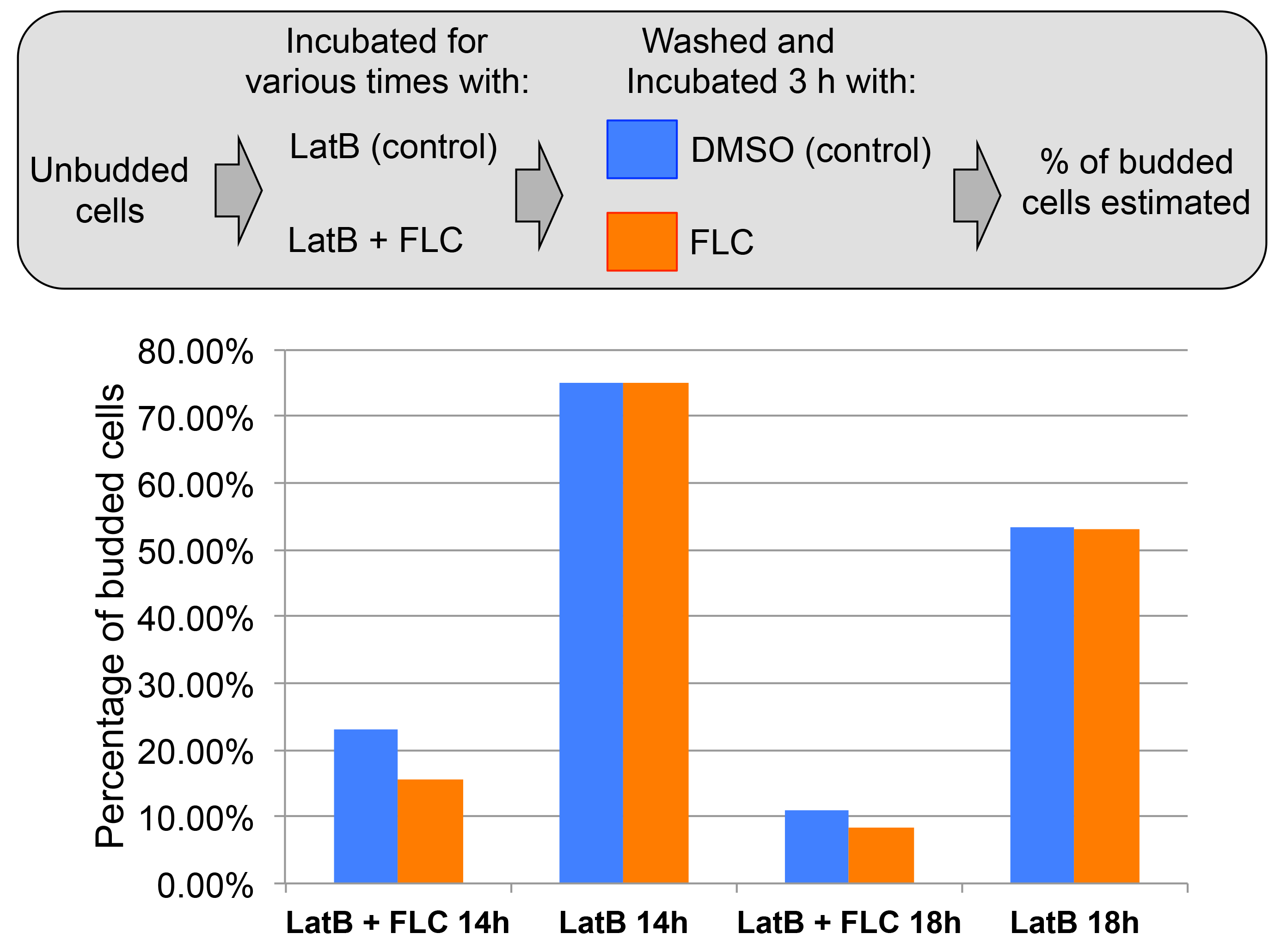

Supplement: FIG S1 [file sph003172297sf1.tif]

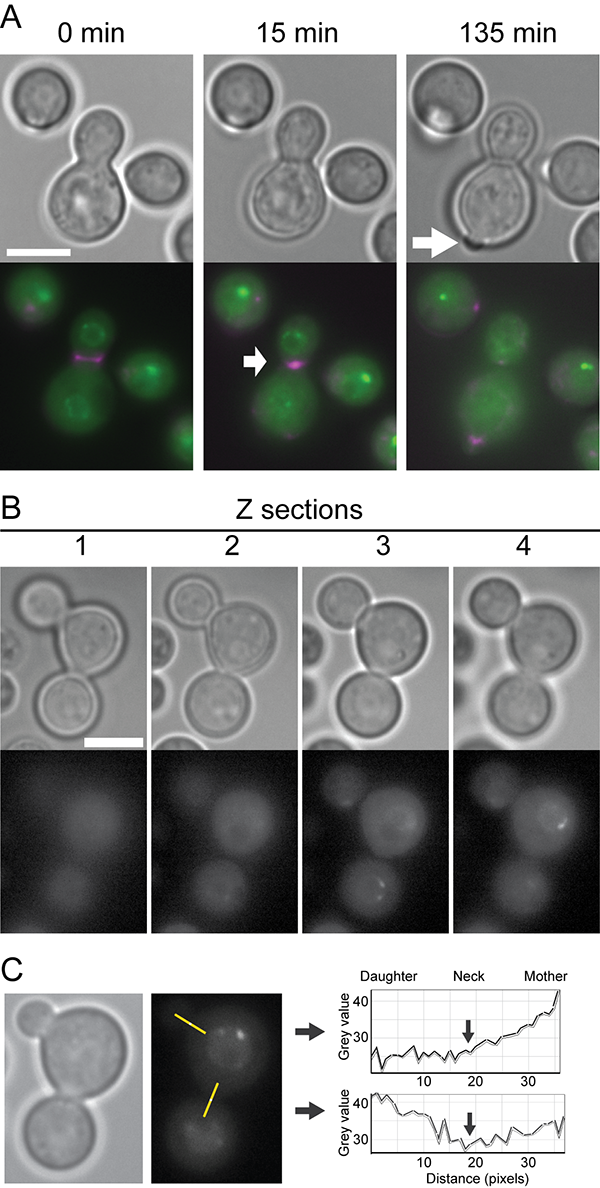

Supplement: FIG S2 [file sph003172297sf2.tif]

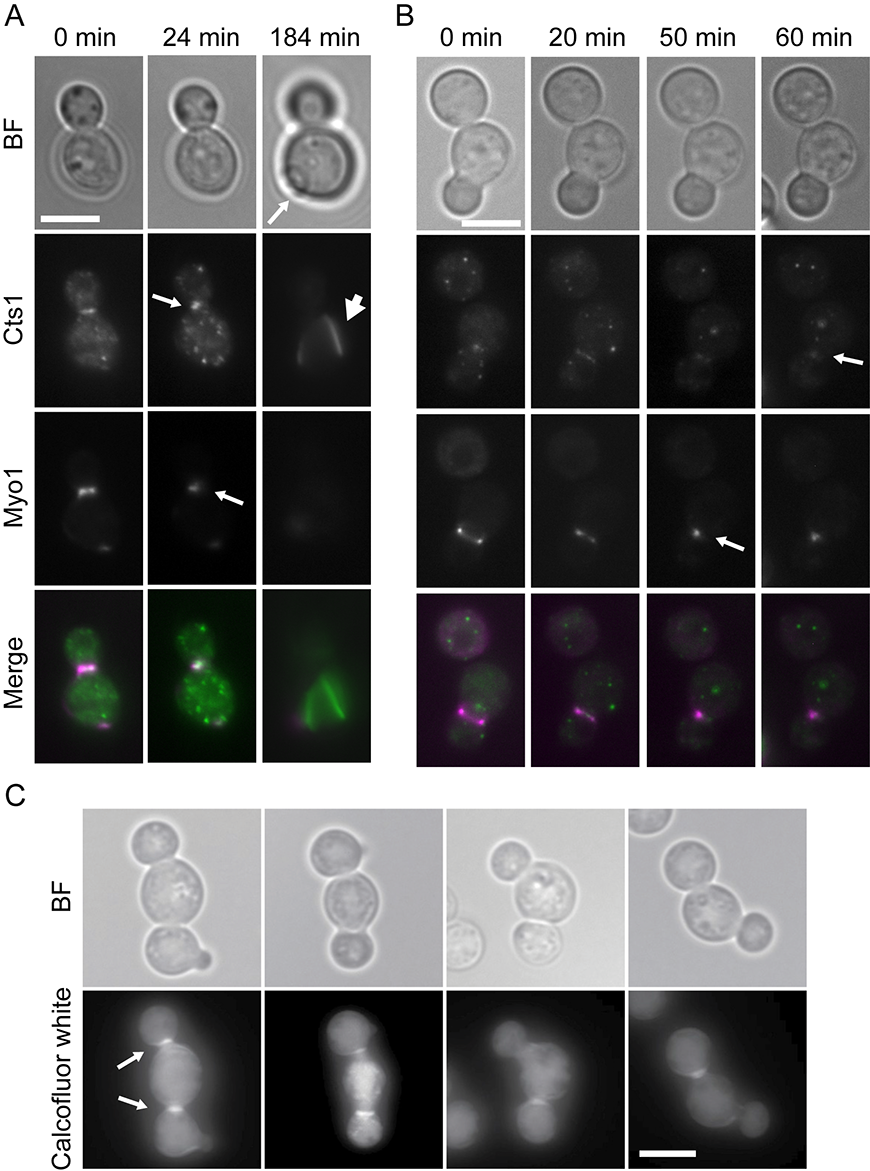

Supplement: FIG S3 [file sph003172297sf3.tif]

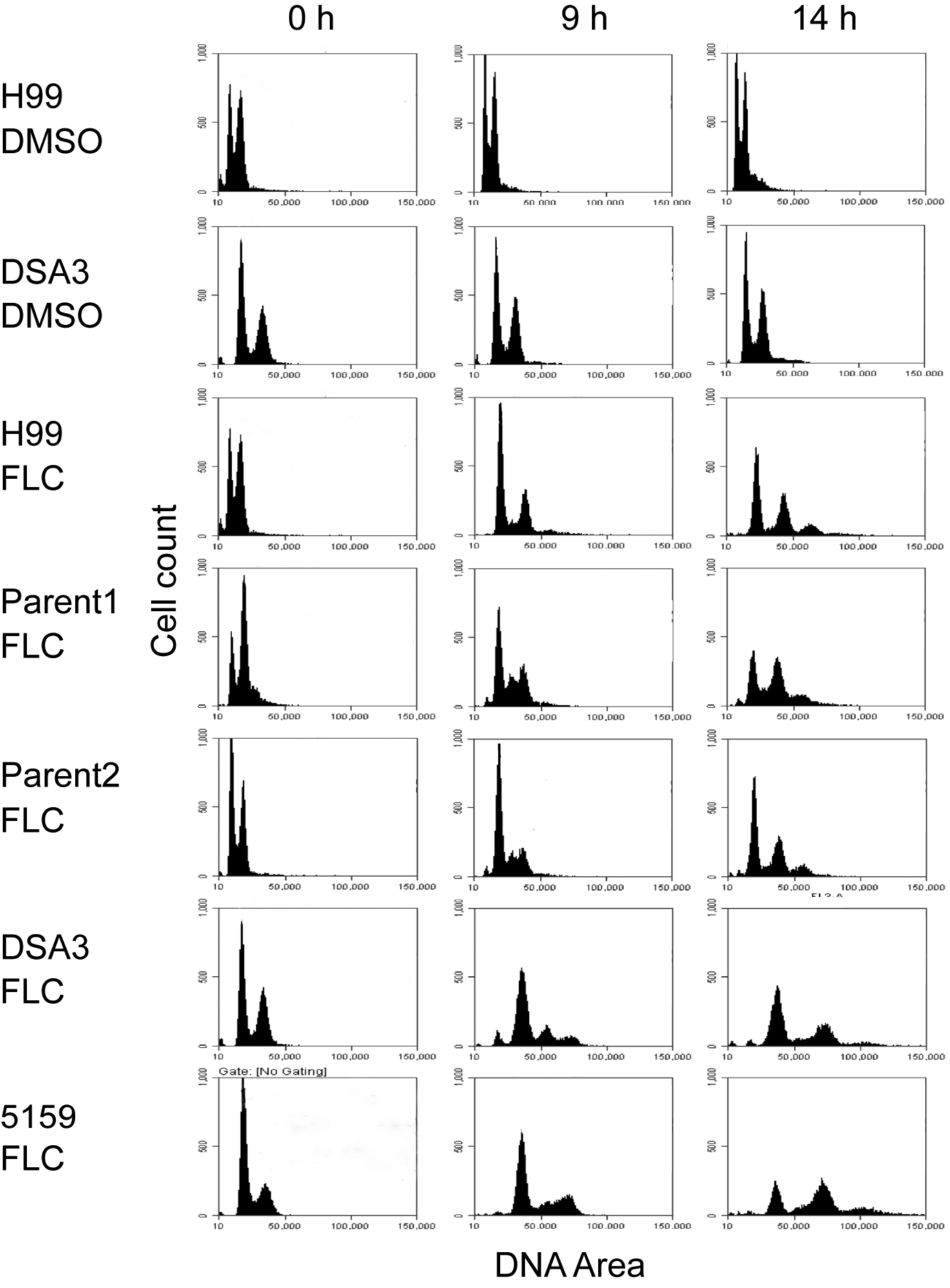

Supplement: FIG S4 [file sph003172297sf4.tif]

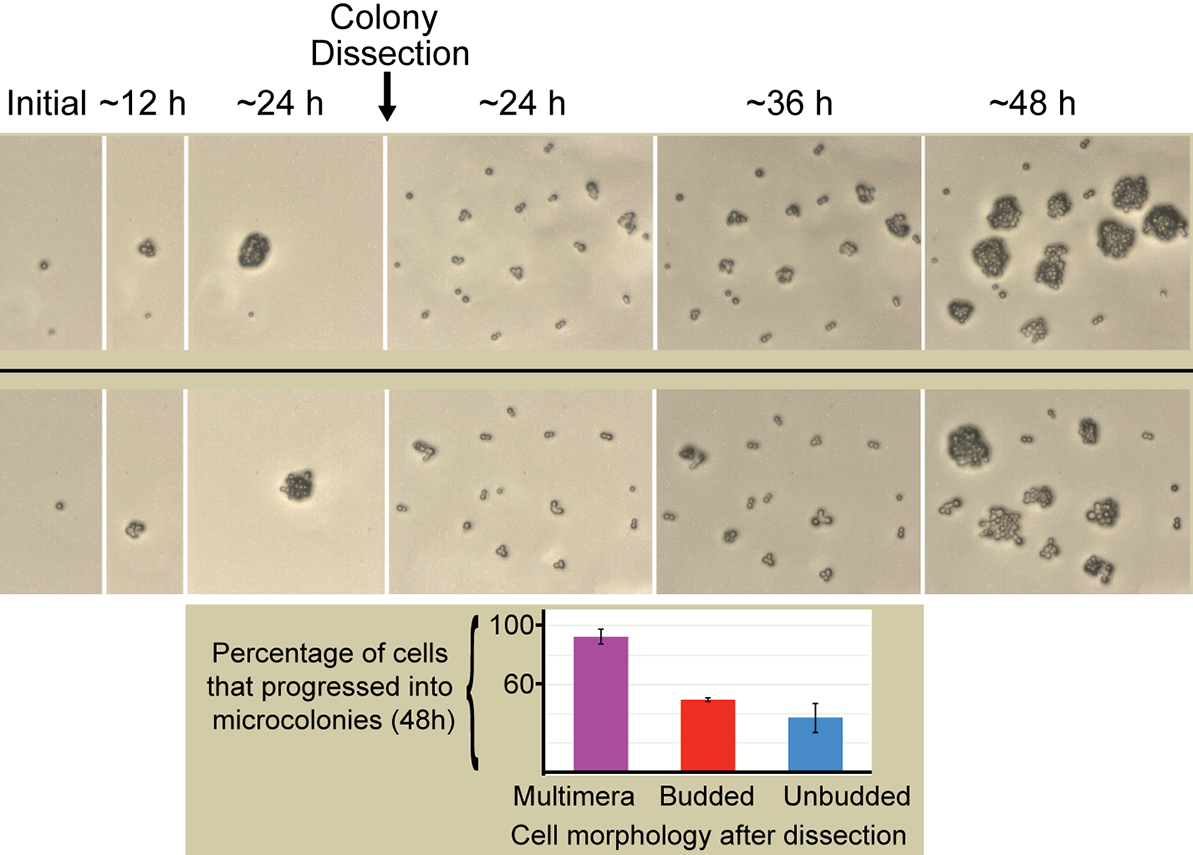

Supplement: FIG S5 [file sph003172297sf5.tif]
